# Combined Bioremediation of Bensulfuron-Methyl Contaminated Soils With Arbuscular Mycorrhizal Fungus and *Hansschlegelia zhihuaiae* S113

**DOI:** 10.3389/fmicb.2022.843525

**Published:** 2022-02-28

**Authors:** Yingying Qian, Guoqiang Zhao, Jing Zhou, Huazhu Zhao, Thamer Y. Mutter, Xing Huang

**Affiliations:** ^1^Department of Microbiology, College of Life Sciences, Nanjing Agricultural University, Nanjing, China; ^2^Department of Biology, College of Science, University of Anbar, Ramadi, Iraq

**Keywords:** *Hansschlegelia zhihuaiae* S113, combined bioremediation, bensulfuron-methyl, arbuscular mycorrhizal fungi, 16s sequencing

## Abstract

Over the past decades, because of large-scale bensulfuron-methyl (BSM) application, environmental residues of BSM have massively increased, causing severe toxicity in rotation-sensitive crops. The removal of BSM from the environment has become essential. In this study, the combined bioremediation of the arbuscular mycorrhizal fungi (AMF) *Rhizophagus intraradices* and BSM-degrading strain *Hansschlegelia zhihuaiae* S113 of BSM-polluted soil was investigated. BSM degradation by S113 in the maize rhizosphere could better promote AMF infection in the roots of maize, achieving an infection rate of 86.70% on the 36th day in the AMF + S113 + BSM group. Similarly, AMF enhanced the colonization and survival of S113 in maize rhizosphere, contributing 4.65 × 10^5^ cells/g soil on the 15th day and 3.78 × 10^4^ cells/g soil on the 20th day to a population of colonized-S113 (based possibly on the strong root system established by promoting plant-growth AMF). Both S113 and AMF coexisted in rhizosphere soil. The BSM-degrading strain S113 could completely remove BSM at 3 mg/kg from the maize rhizosphere soil within 12 days. AMF also promoted the growth of maize seedlings. When planted in BSM-contaminated soil, maize roots had a fresh weight of 2.59 ± 0.26 g in group S113 + AMF, 2.54 ± 0.20 g in group S113 + AMF + BSM, 2.02 ± 0.16 g in group S113 + BSM, and 2.61 ± 0.25 g in the AMF group, all of which exceeded weights of the control group on the 36th day except for the S113 + BSM group. Additionally, high-throughput sequencing results indicated that simultaneous inoculation with AMF and strain S113 of BSM-polluted maize root-soil almost left the indigenous bacterial community diversity and richness in maize rhizosphere soil unaltered. This represents a major advantage of bioremediation approaches resulting from the existing vital interactions among local microorganisms and plants in the soil. These findings may provide theoretical guidance for utilizing novel joint-bioremediation technologies, and constitute an important contribution to environmental pollution bioremediation while simultaneously ensuring crop safety and yield.

## Introduction

Because of their high efficiency, wide spectrum, and low toxicity, sulfonylurea herbicides are among the most widely used herbicides in the world for the control of field weeds. They inhibit catalytic activity of acetolactate synthase (ALS) in the biosynthesis pathway of branched-chain amino acids (such as valine, leucine, and isoleucine) in plants (Saeki and Toyota, 2004). Bensulfuron-methyl (BSM), a representative sulfonylurea herbicide, is usually employed for controlling broadleaf weeds in crop fields. It persists in soil at a half-life of 62 days (yellow-brown soil) or 129 days (yellow fluvo-aquic soil) and is poorly photodegraded (Delgado-Moreno et al., 2007). Unfortunately, their excessive and persistent usage has seriously damaged the natural environment, causing herbicide toxicity in the subsequent rotation of BSM-sensitive crops, changing of the indigenous microbial community structure or richness, and polluting water resources. Therefore, the systematic remediation of residual BSM in soil or water environments is essential to ensure crop safety and to further enhance agricultural yield.

Bioremediation techniques are highly efficient, eco-friendly, low cost, and are heralded as promising tools for the degradation of pollutants that remain in the environment (Pang et al., 2020; Zhang W. et al., 2020). Microbial degradation is a popular and accepted method for the removal of residual BSM from soil. Recently, several microorganisms, including fungi and bacteria, capable of degrading BSM have been reported, such as *Penicillium pinophilum* strain BP-H-02 (Peng et al., 2012), *Methylopila* sp. DKT (Duc and Oanh, 2020), *Bacillus megaterium* L1 (Lin et al., 2010), *Brevibacterium* sp. BH (Zhu et al., 2005) and *Bacillus subtilis* YB1 (Zhang Z. et al., 2020). The main focus of research has been the isolation of BSM-degrading strains, the identification of degradation pathways and products, and detoxification mechanisms of functional enzymes (Wang et al., 2019). In addition, the development of BSM-degrading bacterial inoculums or detoxified-enzyme products has also attracted research attention (Thouand et al., 2011). Nevertheless, the detoxification effect of pure cultures for BSM-contaminated environments (i) is restricted by the natural environmental conditions in the application process of polluted farmland soil, (ii) the survival of degrading strains cannot be maintained for an extended period, and (iii) the effects of a single population are restricted by the metabolism of toxic pollutants. Thus, a mixed community of microorganisms may constitute an alternative strategy for the degradation and removalS113 of BSM, and associations of fungi with bacteria are particularly promising. However, studies on the bioelimination of BSM by multiple microorganisms are rare.

Arbuscular mycorrhizal fungi (AMF), the most widely distributed type of symbionts in soils, and terrestrial plants have adopted an important and mutually beneficial symbiotic relationship. Previous research showed that the extension of the external mycelium of AMF can increase the nutrient absorption and biomass in plants and crops (Bowles et al., 2016; Brundrett and Tedersoo, 2018), including mineral nutrients and water (Leake et al., 2004; Parniske, 2008). The underlying causes are high-affinity inorganic phosphate (Pi) or nitrogen (N) transporters (Bonfante and Genre, 2010), as well as the increased productivity and quality of crops because of the control of pathogens (Baum et al., 2015; Li et al., 2019). Nakmee et al. (2016) also found that inoculation with AMF can produce the greatest biomass, grain dry weight, and total nitrogen uptake in sorghum shoots. Additionally, evidence indicates that AMF can also facilitate plants or crops’ tolerance to potentially toxic elements (PTEs) and adverse environments, such as heavy metals, radioactive elements, or low temperature (Liu et al., 2014; Qiao et al., 2015). This is achieved by mediating the interaction between PTEs and plant roots (Yang G.W. et al., 2015) or alterations in H_2_O_2_ accumulation and ATPase activity. Hence, inoculation with AMF is an effective method for promoting the growth of sensitive crops grown in soils polluted with toxic substances. Furthermore, previous reports suggested that AMF can also enhance the degradation efficiency of a variety of pollutants in soil, including polychlorinated biphenyls and atrazine (Huang H. et al., 2007; Qin et al., 2014). Moreover, these effects also provide ideas and a foundation for combining inoculations of AMF with other biofertilizers or biodegradation agents.

Previous studies merely focused on the degradation of BSM by a pure strain or microbial communities. Similarly, one or limited number of AMF species were used to promote plant growth or the resistance to harsh environments. Nevertheless, a limited number of studies have assessed how herbicide-degrading strains and other functional bacteria or fungi (such as AMF) jointly remediate soils contaminated by organic pollutants. In the present study, the bacterial strain *Hansschlegelia zhihuaiae* S113, capable of degrading BSM into herbicidal-inactive acid by catalyzing de-esterification (Huang X. et al., 2007; Hang et al., 2012), was selected as herbicide-degrading strain. In addition, the AMF *Rhizophagus intraradices*, which markedly improves the transport and uptake of P and water under drought stresses (Li et al., 2014; Leyva-Morales et al., 2019), is added as a plant growth-promoting strain.

The objectives of the present study are to (i) assess the remediation effect of AMF and strain S113 on BSM-contaminated soil, and (ii) discuss the ecological effect of AMF and strain S113 on the remediation of BSM-contaminated soil. The combined bioremediation with AMF and an herbicide-degrading bacterial strain provides a promising remediation technique for the removal of residual herbicides from soils with the combined promotion of plant growth.

## Materials and Methods

### Chemicals, Strains, and Media

BSM (analytical grade, purity > 99%) was purchased from J&K Scientific Company, Ltd (Beijing, China). Cleanert HXN SPE column was purchased from Agela Technologies Co., Ltd., Tianjin, China. HPLC-grade acetonitrile, dichloromethane, and methanol were purchased from Sigma Aldrich, United States. All other reagents used in this study were of analytical grade or higher purity.

*Hansschlegelia zhihuaiae* S113 (Huang X. et al., 2007) was preserved in our laboratory. The AMF *Rhizophagus intraradices* was acquired from the Bank of Glomeromycota in China (BGC) of Beijing Academy of Agriculture and Forestry Sciences, China. Tryptone-yeast (TY) medium for strain S113 cultivation consisted of tryptone (5.0 g/L), yeast extract (3.0 g/L), and CaCl_2_ (0.01 g/L). Mineral salts medium (MSM) contained (g/L): 1.0 NaCl, 1.5 K_2_HPO_4_, 0.5 KH_2_PO_4_, 1.0 NH_4_NO_3_, and 0.2 MgSO_4_**.**7H_2_O. For solid media, 18.0 g of agar was added per liter.

Phosphate-buffered saline (PBS) buffer (pH 7.2) contained (g/L): 8.0 NaCl, 0.2 KCl, 1.42 Na_2_HPO_4_, and 0.27 KH_2_PO_4_. The BSM from rhizosphere soil was extracted with a mixture solution of PBS and acetonitrile (8:2, v:v).

### Soil and Maize Seed Germination

The basic properties of soil used in this experiment are summarized in the following: pH 6.86, organic matter 12.8 mg/kg, available P 6.45 mg/kg, available K 105.48 mg/kg, nitrate-nitrogen 1.77 mg/kg, and ammonium nitrogen 6.24 mg/kg. Soil for the cultivation of maize was air-dried at room temperature and then sieved to a size of 2 mm. The maize seeds of Meiyu 5 (Jiangsu Mingtian Seed Co. Ltd., Nanjing, China) were surface sterilized with 10% H_2_O_2_ solution for 5 min, rinsed repeatedly using sterilized water until H_2_O_2_ was completely removed, and then soaked overnight in sterilized water. The water-swollen seeds were transferred to a glass culture dish covered with two layers of sterile, wet gauze at the bottom, and then left to germinate in the dark for three days. Maize seedlings with nearly equal lengths of root and stem were selected for further study (Zhang et al., 2018).

### Preparation of Arbuscular Mycorrhizal Fungi Inoculant and S113 Suspension

Prior to propagation, pre-incubated soils were autoclave sterilized at 121°C for 2 h to remove indigenous AMF propagules and other microorganisms (Chang et al., 2018). AMF was firstly propagated on the host plant maize planted in a soil-sand mixture (sterilized soil: sand = 3:1) for 4 months. Then, this soil (containing spores, mycelia, and maize root segments) was collected for the preparation of AMF spore suspension, and their concentration was calculated using a hemocytometer. Each milliliter of inoculum contained approximately 9.43 × 10^4^ spores. Each maize seedling root was inoculated with 200 μL spore suspension.

The preparation of strain S113 suspension was performed as described by Zhang et al. (2018) with minor adjustments. Briefly, strain S113 was grown in TY medium at 30°C on a rotary shaker (150 rpm). Cells were harvested in the late log growth phase by centrifugation at 4°C and 5000 *g* for 10 min, washed twice, and then resuspended in sterile water. The final concentration of optical cell density at 600 nm (OD_600_) was adjusted to 1.5 for further investigation.

### Remediation of Bensulfuron-Methyl-Contaminated Soil and Removal of Phytotoxicity to Maize by Arbuscular Mycorrhizal Fungi and Strain S113

Maize seedlings were prepared as described above, and then sown into soils with 0.1, 0.5, 1, 2, 3, and 5 mg/kg of BSM to test appropriate pollution levels, respectively. The growth of maize seedlings at different concentrations was compared after 10 days. To assess the most suitable inoculation amount of strain S113 used for repairing BSM-contaminated soil, a variable volume of S113 suspension (2, 5, 10, 15, and 25 mL) was inoculated into the roots of maize seedlings by root irrigation. Then, all pots were placed in an illumination incubator for 10 days under the following conditions: 14 h of light at 28°C and 10 h of dark at 20°C. ddH_2_O of the same volume instead of S113 suspension served as control. All treatments were carried out in triplicate.

To explore cooperative remediation of BSM-polluted soil by AMF and strain S113, seven treatments and one control were set as follows: (A) control (without any addition), (B) strain S113, (C) BSM, (D) AMF (*Rhizophagus intraradices*), (E) S113 + BSM, (F) AMF + BSM, (G) S113 + AMF, and (H) S113 + AMF + BSM. Maize seedlings were randomly collected to measure the growth parameters, as well as to visualize the infection of AMF on maize roots at 12 d, 24 d, or 36 d. In addition, rhizosphere soil was also collected every 6 days to measure the residual concentration of BSM and to extract the total DNA of soil for studying the microbial community in maize rhizosphere soil. Each treatment was replicated three times. The degradation experiment refers to Huang X. et al. (2007).

### Determination of Arbuscular Mycorrhizal Fungi Infection Rate

Total root systems of maize from each treatment were cut into 1 cm pieces and fixed in FAA solution (13 mL formaldehyde, 5 mL acetic acid, 200 mL 50% ethanol). Fixed roots were heated at 90°C for 1 h in 10% potassium hydroxide (KOH) solution. The roots were rinsed with water and acidified with 2% HCl for about 5 min, then stained at 90°C for 30 min using 0.05% trypan blue in a mixture solution of lactic acid, glycerol, and distilled water (1:1:1, v:v:v) (Phillips and Hayman, 1970). Finally, entire roots were soaked in decoloring solution (50 mL lactic acid, 100 mL glycerin, and 50 mL distilled water) overnight. Subsequently, 30 root segments were selected randomly and their AMF colonization was observed under an ordinary optical microscope at 200 × magnification. The mycorrhizal colonization rate was estimated using the method of Biermann and Linderman (1981).

### Effects of Arbuscular Mycorrhizal Fungi on the Colonization of Strain S113

To facilitate colonization observation, strain S113-*gfp* (containing reporter genes *gfp*) was constructed as previously described (Zhang et al., 2018). Colonization on the root surface was detected by confocal laser scanning microscope (CLSM, Leica TCS SP3). The maize seedlings, prepared as described in Section “Soil and maize seed germination,” were irrigated by S113-*gfp* (OD_600_ = 1.5, 15 mL), S113-*gfp* (OD_600_ = 1.5, 15 mL) + AMF (200 μL), or sterile water of the same volume, then incubated in a growth chamber for 25 days. The seedlings were gently pulled out of pots at an interval of five days to observe the colonization of S113 on the roots. In addition, root and rhizosphere soil samples were collected for the quantitative determination of S113 colonization. The total DNA of both rhizosphere soil and maize roots were extracted using the FastDNA^®^ SPIN Kit for Soil (MP Biomedicals™) and plant DNA extraction kit (Aidlab Biotechnologies Co., Ltd., Beijing, China), respectively.

The S113 colonization in maize root or rhizosphere soil was detected by quantitative real-time PCR (qPCR). The qPCR reaction solution contained 10.0 μL of 2 × ChamQ Universal SYBR qPCR Master Mix (Vazym), 0.4 μL of *sulE*-F/R (10 μM), 2.0 μL of template DNA (from maize root or rhizosphere soil), and 7.2 μL double distilled water. qPCR was performed in a QuantStudio 6 Flex system using the following procedure: 95°C for 3 min, followed by 40 cycles of 95°C for 5 s, and 60°C for 34 s. DNA standard curves were prepared as previously described (Chi et al., 2013) using the known gene *sulE*, encoding a de-esterification esterase that degraded BSM in the genome sequence of strain S113, because it is a single-copy gene and has low sequence similarities (44% identity) (Zhang et al., 2018). Briefly, the partial sequence of gene *sulE* (202 bp) amplified from S113 genomic DNA with the primers *sulE*-F (5′-CGCCAAGAACGTGAGGGGGAT-3′) and *sulE*-R (5′-CTGTGAGCGTAGCGAGTGACT-3′) was cloned into the vector pMD19-T (TaKaRa, Beijing, China). Plasmids (pMD19-T/s*ulE*) were extracted from recombinants and used as standards for quantitative analyses. The concentration of recombinant plasmid DNA was determined by NanoDrop 2000/2000c (ThermoFisher Scientific, Wilmington, DE, United States). Ten-fold serial dilutions of known concentrations of the plasmid DNA were used as template for qPCR to generate an external standard curve.

### Soil Bacterial Community Analyses

Extracts of total DNA of rhizosphere soil were obtained from eight group samples (see Section “Effects of AMF on the colonization of strain S113” for DNA extraction). The concentration of DNA in samples containing visible bands was assessed using NanoDrop 2000/2000c before sequencing. The quality of soil total DNA was tested via 1% agarose gel electrophoresis. The hypervariable V_4_–V_5_ fragment of the 16S rRNA gene was amplified by the universal primer pair 338F (5′-ACTCCTACGGGAGGCAGCA-3′)/806R (5′-ACTCCTACGGGAGGCAGCA-3′) with barcode. PCR reactions were carried out in 30 μL reactions with 15 μL of Phusion^®^ High-Fidelity PCR Master Mix (New England Biolabs), using 0.2 μM of forward and reverse primers, and 10 ng template DNA. Thermal cycling consisted of initial denaturation at 98°C for 1 min, followed by 30 cycles of denaturation at 98°C for 10 s, annealing at 50°C for 30 s, and elongation at 72°C for 30 s. Finally, 72°C for 5 min. PCR products were assessed via 2% agarose gel electrophoresis and purified the bright main strip between 400 and 450 bp with GeneJET Gel Extraction Kit (Thermo Scientific). Purified PCR products were mixed for generating sequencing libraries using NEB Next^®^ Ultra™ DNA Library Prep Kit for Illumina (NEB, United States) following the manufacturer’s recommendations. The library quality was assessed with Qubit@ 2.0 Fluorometer (Thermo Scientific) and Agilent Bioanalyzer 2100 system. The library was sequenced on an Illumina HiSeq platform and 250 bp paired-end reads were generated, then merged using FLASH (Magoč and Salzberg, 2011). Briefly, reads were firstly filtered by QIIME quality filters. Then, sequences with ≥ 97% similarity were assigned to the same operational taxonomic units (OTUs). A representative sequence for each OTU was chosen to annotate taxonomic information using the RDP classifier (Wang et al., 2007).

### Chemical Analysis

For extraction of BSM from rhizosphere soil, a 10 g rhizosphere soil sample was extracted with 20 mL mixed PBS and acetonitrile solution, then shocked at 150 rpm for 1 h, and centrifuged at 8000 *g* for 10 min. The extraction process was repeated three times for every sample and all supernatants were collected and acidified to a pH of 2.5. A Cleanert HXN cartridge was used to purify supernatants. The elution solution was air-dried, then resuspended in 1 mL methanol, and the residual BSM was measured by high-performance liquid chromatography (HPLC) (Dionex UltiMate 3000) equipped with a C_18_ reverse-phase chromatographic column (4.6 × 250 mm, 5 μm, Agilent Technologies, Palo Alto, CA, United States), using a UV detector at 230 nm. Water:acetonitrile:acetic acid (40:60:0.5, v:v:v) was used as the mobile phase at a flow rate of 1 mL/min for detection. The concentration was determined via comparison against values in the calibration curve.

### Statistical Analysis

Data were analyzed by one-way ANOVA and Duncan’s test to compare means. *p* values < 0.05 were considered to represent statistically significant differences. Statistical data were analyzed using GraphPad Prism v8.0.2.263 software (San Diego, CA, United States).

### Data Availability

All of the sequencing data involved in this manuscript had been deposited in the NCBI database (BioProject ID:PRJNA793182), and could be download from the link: https://www.ncbi.nlm.nih.gov/sra?linkname=bioproject_sra_all&from_uid=793182.

## Results

### Promotion of S113 on Arbuscular Mycorrhizal Fungi Infection to the Maize Root System

**FIGURE 1:**
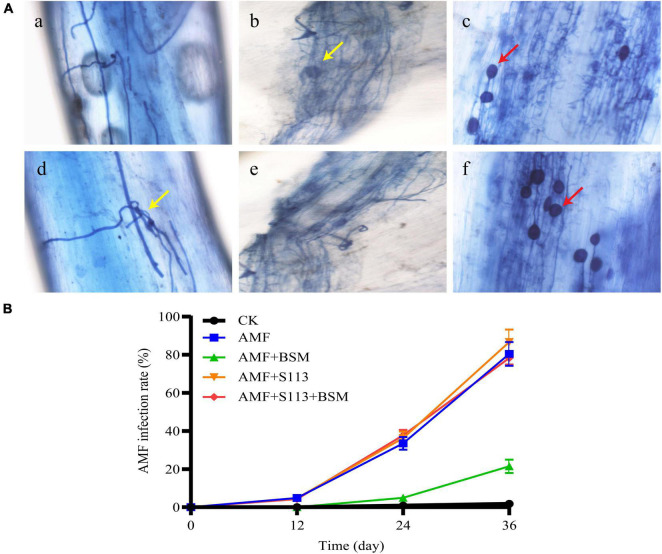
Infection of the Arbuscular mycorrhizal fungi (AMF) to the maize root system. **(A)** *Rhizophagus intraradices* colonization in maize roots, as observed under an optical microscope (200 ×). (a) (d) Root segment with low infection rate; (b) (e) Root segment with moderate infection rate; (c) (f) Root segment with high infection rate. The red arrows indicate spores; the yellow arrows indicate hyphae or mycelium. **(B)** The infection rate of AMF to maize root system under different treatments.

Photomicrographs of the root segments of maize infected by AMF are shown in Figure 1A. Typical structures of AMF and plant symbiosis, such as arbuscular hyphae, mycelium, and spores, were distinctly observed by an ordinary optics microscope. These structures indicate a friendly symbiotic relationship between AMF and maize roots.

The maize roots were AMF infected to a certain extent (Figure 1B). On the 12th day, the lower infection rates of 4.83%, 4.80%, and 4.30% were observed in the treatment groups D (AMF), G (S113 + AMF), and H (S113 + AMF + BSM), respectively. However, the AMF infection rates in groups D, G, and H on the 24th day reached 33.50%, 36.26%, and 37.76%, respectively, while the infection rate of group F (AMF + BSM) was only 4.93%. This may be the result of BSM causing serious toxicity to the maize root system, which in turn may have affected AMF infection. With the inoculation of BSM-treated soil with BSM-degrading strain S113, the AMF infection rate markedly increased to about 78.30% on the 36th day (Figure 1B). The possible reason for the significant increase of AMF infection rate after inoculation of S113 degrading bacteria is that strain S113 removes BSM in rhizosphere soil and reduces the formation of phytotoxicity. Only 21.50% of the infection rate was observed in the BSM-amended soil on the 36th day (Figure 1B), which still showed a significant difference from the control.

### Increase of Strain S113 Colonization by Arbuscular Mycorrhizal Fungi

**FIGURE 2:**
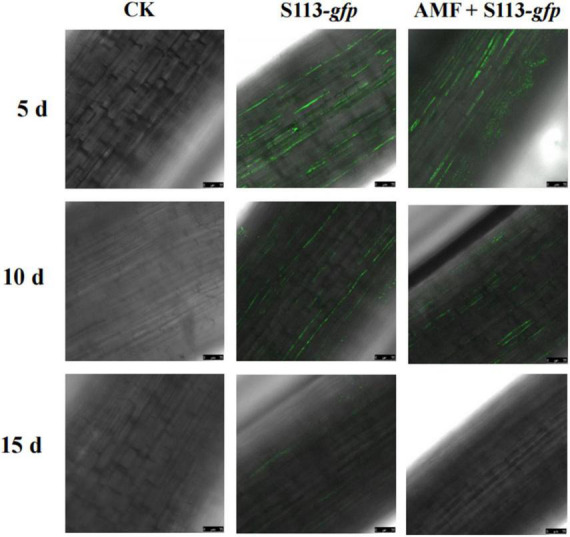
Confocal laser scanning microscope images of S113 colonization on maize roots. The treatment was irrigated with S113 or S113 + Arbuscular mycorrhizal fungi (AMF). The control was treated with sterile water.

To assess the colonization of rhizosphere microorganisms on the root surface and rhizosphere of crops, a plasmid carrying the *gfp* gene was introduced into strain S113. The cells of strain S113 strongly colonized the surface of corn roots, which could be observed under a confocal laser scanning microscope (CLSM) (Figure 2). The most apparent fluorescence intensity in maize root could be seen after irrigating S113-*gfp* on the 5th day. However, the colonization effect of strain S113-*gfp* gradually weakened after the 10th day. This may be the result of a gradual decline in the growth activity of strain S113-*gfp* during maize cultivation.

**FIGURE 3:**
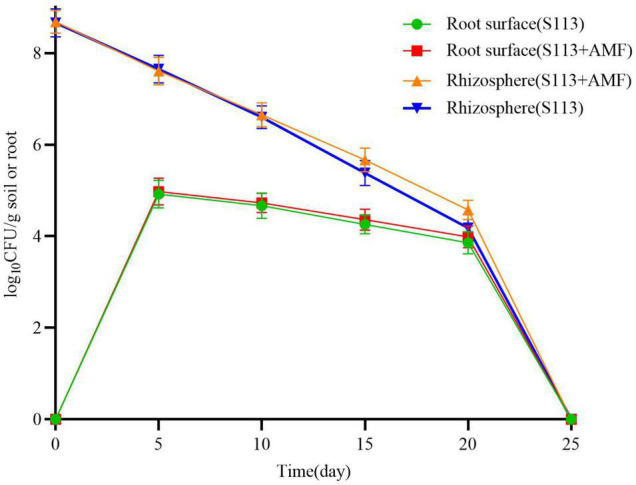
Effects of Arbuscular mycorrhizal fungi (AMF) on the colonization of S113 in the rhizosphere and root surface. Dynamic of S113 population on roots surface and in the rhizosphere of maize under different treatments. The data are shown as log_10_CFU per gram soil or roots. The error bars indicate standard deviations for three samples.

According to the standard curve, strain S113 colonizing the maize root surface and rhizosphere was quantified by qPCR (Figure 3). The initial density of strain S113 colonizing rhizosphere soil reached 4.7 × 10^8^ cells/g soil. The number of S113 in the rhizosphere gradually decreased with prolonged colonization time, and the population of strain S113 colonizing the corn root surface rapidly increased. On the 5th day, S113 reached maximum colonization (9.44 × 10^4^ cells/g root) on the root surface. This result matches CLSM images of S113 colonization on maize roots (Figure 2). The amounts of strain S113 of 4.65 × 10^5^ cells/g soil on the 15 days and 3.78 × 10^4^ cells/g soil on the 20 days, colonized in the rhizosphere soil were higher compared with the treatment without AMF inoculation (2.39 × 10^5^ cells/g soil on the 15 days and 1.52 × 10^4^ cells/g soil on the 20 days). These results suggest that inoculation of AMF slightly promotes S113 colonization on maize root surface or rhizosphere soil.

### Bioremediation of Bensulfuron-Methyl-Polluted Soil by the Combination of Arbuscular Mycorrhizal Fungi and S113

The appropriate inoculum of strain S113 and BSM contamination concentration were determined before assessing the elimination of the BSM phytotoxicity in maize by S113 and/or AMF. A variety of BSM concentrations caused a certain extent of phytotoxicity in maize seedlings (Supplementary Figure 1A). The leaves of maize seedlings were slightly shriveled, purple, and without moisture in 5 mg/kg BSM-contaminated soil, while maize seedlings did not wither after exposure to 3 mg/kg BSM. Therefore, a BSM concentration of 3 mg/kg was used to cultivate maize in subsequent pot experiments. Correspondingly, the strain S113 capable of biological repair could partially alleviate phytotoxicity to maize. Using > 15 mL suspension of strain S113 with OD_600_ = 1.5 irrigated to maize roots grown in 3 mg/kg BSM-contaminated soil achieved recovery of maize growth compared to control (Supplementary Figure 1B). Thus, a 15 mL suspension of strain S113 (OD_600_ = 1.5) was selected to inoculate the maize roots for all following remediation experiments.

**FIGURE 4:**
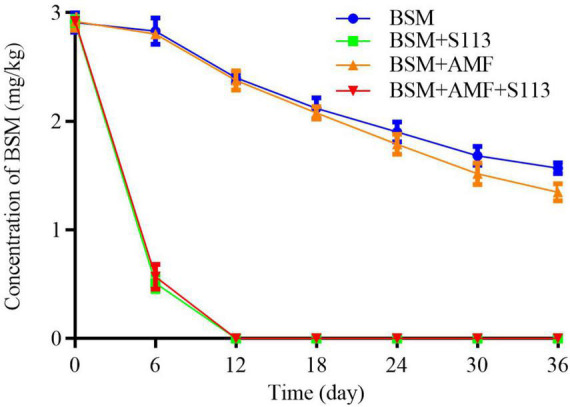
The residual concentration of Bensulfuron-methyl (BSM) in Rhizosphere soil.

The residual amount of BSM in rhizosphere soil is shown in Figure 4. The inoculation of strain S113 could notably reduce the concentration of BSM in rhizosphere soil on the 6th day (from 3 to about 0.5 mg/kg). Furthermore, the residual BSM could no longer be detected after 12 days. Nevertheless, the concentration of BSM in BSM + S113 treatment and BSM + S113 + AMF treatment did not show a significant difference during the entire remediation process of BSM contaminated soil. Removal of BSM in group F was still faster than the treatment without AMF (i.e., group C). The residual BSM concentrations were 1.90 mg/kg and 1.78 mg/kg soil in treatments C and F on the 24th day and 1.57 mg/kg and 1.35 mg/kg soil on the 36th day. This showed that AMF addition has a certain promoting effect on BSM degradation in soil.

### Elimination of Bensulfuron-Methyl Phytotoxicity to the Maize by Combining Arbuscular Mycorrhizal Fungi and S113

**FIGURE 5:**
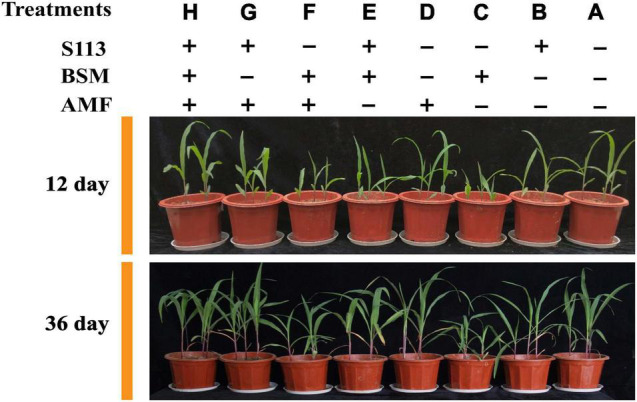
The growth of maize under different treatments. (A) control (CK); (B) S113; (C) Bensulfuron-methyl (BSM); (D) Arbuscular mycorrhizal fungi (AMF); (E) S113 + BSM; (F) AMF + BSM; (G) S113 + AMF; (H) S113 + AMF + BSM. **+**: addition; **−**: without addition.

**FIGURE 6:**
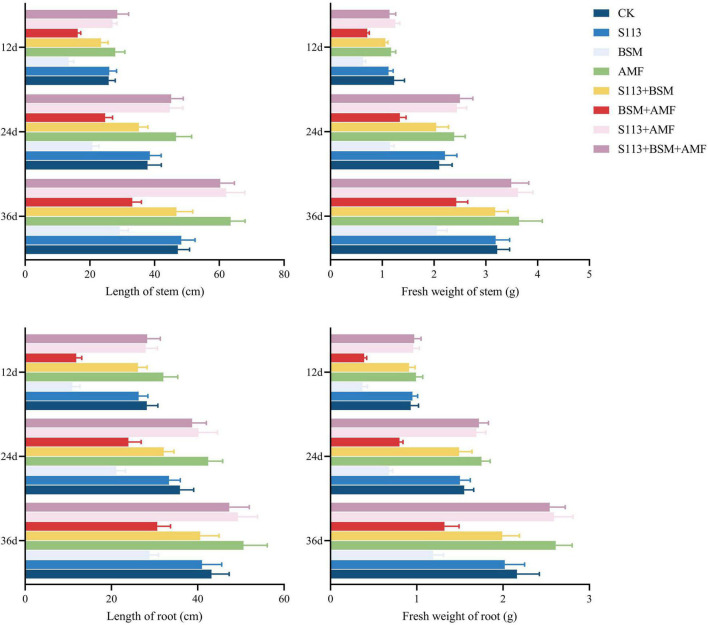
The growth of maize under different treatments. CK: the seedlings without Bensulfuron-methyl (BSM) and S113 or Arbuscular mycorrhizal fungi (AMF); S113: seedlings were inoculated with S113 by root irrigation; BSM: soils were treated with bensulfuron-methyl, 3.0 mg/kg; AMF: seedlings were inoculated with AMF spore suspension (200 μL); S113 + BSM: seedlings were treated with BSM and inoculated with S113; AMF + BSM: seedlings were treated with BSM and inoculated with AMF spore suspension; S113 + AMF: seedlings were inoculated with S113 and AMF spore suspension; S113 + BSM + AMF: seedlings were inoculated with S113 and AMF spore suspension, and soils were treated with BSM.

The growth of maize under different treatments is shown in Figures 5, 6. Strain S113 did not affect the growth of maize. The stem length, root length, fresh-stem weight, and fresh-root weight of maize in group C were 13.49 ± 1.28 cm, 10.92 ± 1.29 cm, 0.63 ± 0.05 g, and 0.37 ± 0.05 g on the 12th day, respectively, which were significantly lower than control. In contrast, addition of strain S113 (group E) could notably eliminate BSM phytotoxicity in maize seedlings. The length and fresh weight of stem and root gradually increased to 23.43 ± 2.20 cm, 26.14 ± 2.35 cm, 1.06 ± 0.05 g, and 0.92 ± 0.07 g on the 12th day, respectively, and were comparable to the control level. This indicated that BSM could strongly inhibit the growth of maize without S113 irrigation, and strain S113 could significantly alleviate the phytotoxicity of BSM to maize seedlings.

AMF could not directly degrade BSM from rhizosphere soil (there was no significant difference between BSM + AMF and BSM groups). However, AMF significantly enhanced the growth of maize regardless of whether it was grown in BSM-treated rhizosphere soil, especially S113 + AMF, S113 + AMF + BSM, and AMF. For example, the fresh weight of maize roots of groups S113 + AMF, S113 + AMF + BSM, and AMF was 2.59 ± 0.22 g, 2.54 ± 0.18 g, and 2.61 ± 0.19 g higher, respectively, compared to the CK group on the 36th day. The possible reason is that the lower infection rate still provided the possibility for information exchange between maize roots and AMF.

### Recovery of the Soil Bacterial Community Structure Damaged by S113

**FIGURE 7:**
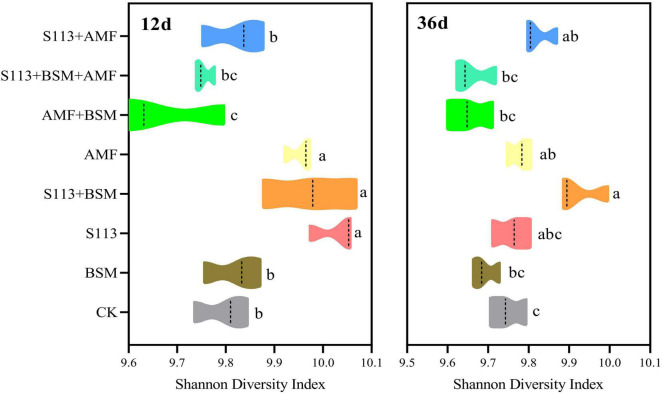
Shannon diversity index for microbial communities of different treatments on the 12th day and 36th day. Note: CK: the seedlings without Bensulfuron-methyl (BSM) and S113 or Arbuscular mycorrhizal fungi (AMF); S113: seedlings were inoculated with S113 by root irrigation; BSM: soils were treated with bensulfuron-methyl, 3.0 mg/kg; AMF: seedlings were inoculated with AMF spore suspension (200 μL); S113 + BSM: seedlings were treated with BSM and inoculated with S113; AMF + BSM: seedlings were treated with BSM and inoculated with AMF spore suspension; S113 + AMF: seedlings were inoculated with S113 and AMF spore suspension; S113 + BSM + AMF: seedlings were inoculated with S113 and AMF spore suspension, and soils were treated with BSM. Values with different letters are significantly different at the *P* < 0.05 according to Duncan’s test.

Shannon diversity indices of different treatment groups were calculated using QIIME software to assess the diversity of the soil community structure. The Shannon diversity of group S113 + BSM presented a significant difference with only the addition of BSM according to the Duncan test on the 12th day (Figure 7). In other words, strain S113 markedly changed the diversity of the community structure of indigenous microorganisms at the beginning of inoculation. However, with the addition of AMF, the diversity of the microbial community structure in maize rhizosphere soil gradually recovered to the level of the native indigenous microorganisms on the 36th day (treatment S113 + BSM + AMF vs BSM) (Figure 7).

**FIGURE 8:**
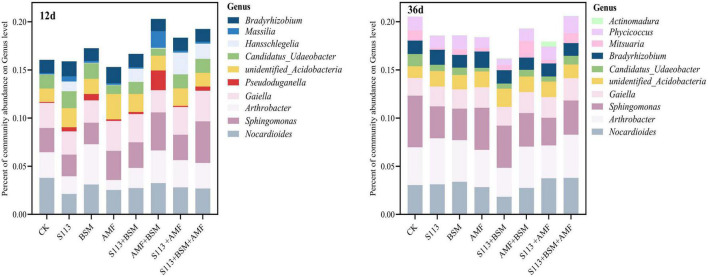
Relative abundance histograms for different treatments in genus level. The top 10 of total species abundance are displayed. Each color represents a different genus level species. CK: the seedlings without BSM and S113 or AMF; S113: seedlings were inoculated with S113 by root irrigation; BSM: soils were treated with bensulfuron-methyl, 3.0 mg/kg; AMF: seedlings were inoculated with AMF spore suspension (200 μL); S113 + BSM: seedlings were treated with BSM and inoculated with S113; AMF + BSM: seedlings were treated with BSM and inoculated with AMF spore suspension; S113 + AMF: seedlings were inoculated with S113 and AMF spore suspension; S113 + BSM + AMF: seedlings were inoculated with S113 and AMF spore suspension, and soils were treated with BSM.

Different treatment conditions strongly impacted the abundance of the bacterial community. *Nocardioides*, *Arthrobacter*, *Sphingomonas*, *Gaiella*, *unidentified_Acidobacteria*, and *Candidatus_Udaeobacte*r had consistently higher relative abundances in all samples over the entire remediation process of contaminated soil (Figure 8). The abundance of genus *Hansschlegelia* increased dramatically on days 12, and a decreasing trend was observed on the 36th day, indicating that strain S113 can survive in the soil for at least 12 d. Interestingly, a lower abundance was found for genus *Nocardioides* in S113 + BSM at any time.

## Discussion

Exploring sustainable and environmentally friendly remediation technologies for herbicide-contaminated soil has received considerable attention. Bioremediation is the most promising biotechnological approach to remove pollution from soil, including sulfonylureas herbicides (Egorova et al., 2017; Bhatt et al., 2020). To date, few microorganisms capable of degrading BSM have been isolated and identified. Prominent examples are *Brevibacterium* sp. (Luo et al., 2008), *Brevibacterium* sp. strain BH (Zhu et al., 2005), *Bacillus megaterium* strain L1 (Lin et al., 2010), *Penicillium pinophilum* strain BP-H-02 (Peng et al., 2012), *Bacillus subtilis* strain YB1 (Zhang Z. et al., 2020), and *Methylopila* sp. strain DKT (Duc and Oanh, 2020). In the present study, the highly efficient BSM-detoxifying strain *Hansschlegelia zhihuaiae* S113 was used for the remediation of BSM from soil, as relevant research showed that this strain achieved promising degradation effect (Huang X. et al., 2007; Hang et al., 2012). This provides valuable degrading-strain resources for the bioremediation of BSM-contaminated soil.

Associations between host plants and mycorrhizal fungi depend on a very close and useful relationship where plants provide nutrients and residency for mycorrhizal fungi, which in turn either directly or indirectly improve the nutrient status of their host plants and convey resistance to environmental stresses (Smith and Read, 2002). However, this symbiotic relationship between plants and AMF is not always beneficial for both. For example, AMF has been shows to also impose adverse effects on the growth of tobacco and sorghum plants (Watts-Williams et al., 2019). The findings of the present study demonstrate that AMF could neither degrade BSM nor improve the degradation effect of strain S113 to BSM in rhizosphere soil (Figure 4). However, it could, to varying degrees, promote the growth of maize whether it was planted in BSM-contaminated soil or not (BSM vs BSM + S113) (Figures 5, 6), which is consistent with a previous report (Yang Y. et al., 2015). Most species of AMF belong to the sub-phylum Glomeromycotina of the phylum Myxomycetes (Spatafora et al., 2016), and already 25 genera have been described in this subphylum (Redecker et al., 2013). The possibility cannot be ruled out that other types of AMF are also capable of assisting the removal of BSM or other herbicides in plants’ root or rhizosphere soil. For example, Huang H. et al. (2007) reported that AMF treatment enhances the degradation efficiency of the herbicide atrazine in soil. Hence, in future experiments, *Rhizophagus intraradices*, which was used in this study, could be replaced with other AMF and effects on the dissipation of BSM in soil by strain S113 can be assessed further.

The chemotaxis and colonization of rhizosphere microorganisms on plant root surfaces and rhizosphere are considered as a precondition for function, such as metabolizing pollutants that exist in the plant rhizosphere (Moore et al., 2006; Weyens et al., 2010). The leek endophyte *Sphingomonas* sp. HJY, which colonizes the roots, stems, and leaves of leek plants, efficiently degraded chlorpyrifos in leek and lets its growth condition gradually return to a healthy level (Feng et al., 2017). The biocontrol bacterium *Bacillus subtilis* CE1 was shown to be capable to inhibit the pathogen *Fusarium verticillioides* on the surface of corn roots. This reduces diseases and promotes corn growth, which has largely been associated with its ability to colonize corn roots (Cavaglieri et al., 2005). *Klebsiella pneumonia* NG14 is also capable to colonize rice roots and participates in nitrogen fixation (Liu et al., 2011). Previous research showed that strain S113 could colonize maize roots by forming a biofilm to promote root exudates. The result of this research showed that on the 5th day, strain S113 reached its maximum colonization of the root surface (Figures 2, 3) and could survive on the root surface or rhizosphere for at least 20 days in the presence of AMF (Figure 3). Moreover, the S113 population living on the maize root surface and rhizosphere increased slightly after AMF inoculation. This may explain why infection with AMF alternated the types and amounts of organic acids secreted by the roots of maize, and further promoted S113 chemotaxis to maize roots (Feng et al., 2017; Shyam et al., 2017). It has been reported that AMF infecting the root surface of plants could efficiently regulate their own colonization and that of other strains by changing the composition of root exudates secreted by plants (Pinior et al., 1999). Another reasonable hypothesis is that AMF infection improved the growth condition of the maize root system, which further promoted the colonization and biofilm formation of strain S113 on the root surface. Overall, the AMF *Rhizophagus intraradices* helped the remediation of BSM-contaminated rhizosphere soil by S113, but also promoted the colonization of BSM-degrading strains and the growth of maize cultivated in BSM polluted soil. In other words, the plant-growth-promoting mycorrhizal fungi and BSM degrading bacteria AMF and S113 coexist and establish a successful interaction in the soil environment. Researching S113 colonization and survival in the presence of AMF in the rhizosphere soil or root surface might provide valuable information or new theoretical guidance for the bioremediation of herbicide-contaminated soil. Simultaneously, this can increase crop yield. However, the specific mechanism of how AMF affects the colonization of other rhizosphere microorganisms in the plant rhizosphere soil still merits further exploration.

In particular, the formation of AMF extra-root mycelia after colonization of the plant roots system can alter the structure and composition of the soil microbial community (Toljander et al., 2007; Luginbuehl and Oldroyd, 2017). In the present study, inoculation with AMF indeed increased the diversity and abundance of maize rhizosphere soil community (treatment AMF vs control). For instance, the α-diversity index of the AMF group was high (*p* < 0.001) during the entire experiment period. The relative richness of the genus *Sphingomonas* increased strongly after AMF addition on the 36th day (Figures 7, 8). Although the formation of the mycorrhizal network with plants in the ecological environment is a key factor affecting the structure of the rhizosphere microbial community (Lekberg et al., 2007), the inoculation of other bacteria or bacterial consortia also affects the community structure. However, the ecological behavior of microbes that degrade chemical herbicides, including their interaction with soil indigenous microorganisms and their response to the herbicide substrates, remains less well defined. The results of high-throughput sequencing of 16S rRNA indicated that inoculation of maize root with BSM-degrading stain S113 had a variable effect on the local microbial diversity and relative abundance at the genus level (Figure 8). However, AMF-S113 combined remediation to the BSM contaminated soils would not influence the soil micro-ecosystem, which represents a major advantage of bioremediation approaches.

The joint remediation of BSM contaminated soil in this study was only carried out in a pot experiment. Thus, the effect of complex natural environmental factors on the combined bioremediation efficiency in the soil environment, as well as the survival rate of herbicide-degrading strains and plant-growth-promoting fungi in agricultural sites, remains to be studied in field applications. Moreover, the molecular mechanism of how strain S113 promotes AMF (*Rhizophagus intraradices*) infection in the maize rhizosphere, as well as the promotion restoration mechanism of the soil bacterial community by AMF, needs further investigation.

## Data Availability Statement

The datasets presented in this study can be found in online repositories. The names of the repository/repositories and accession number(s) can be found below: BioProject, PRJNA793182.

## Author Contributions

YQ designed the work, conducted the experiments, and wrote the manuscript. GZ and JZ statistically analyzed the data and participated in revising the manuscript. HZ conducted the experiments and analyzed the data. XH and TM guided the data analysis and revised the manuscript. All authors contributed to the study and approved the final submitted version.

## Conflict of Interest

The authors declare that the research was conducted in the absence of any commercial or financial relationships that could be construed as a potential conflict of interest.

## Publisher’s Note

All claims expressed in this article are solely those of the authors and do not necessarily represent those of their affiliated organizations, or those of the publisher, the editors and the reviewers. Any product that may be evaluated in this article, or claim that may be made by its manufacturer, is not guaranteed or endorsed by the publisher.
