# Supplementary material for: Combined Bioremediation of Bensulfuron-Methyl Contaminated Soils With Arbuscular Mycorrhizal Fungus and Hansschlegelia zhihuaiae S113
Source: Front Microbiol. 2022 Feb 28;13:843525. doi: 10.3389/fmicb.2022.843525 (PMC8918986; doi:10.3389/fmicb.2022.843525)
Supplement: Supplementary file 1 [file Data_Sheet_1.doc]

**Supporting Information for**

**Combined bioremediation of bensulfuron-methyl contaminated soils with arbuscular mycorrhizal fungus and *Hansschlegelia zhihuaiae* S113**

Yingying Qian^1^, Guoqiang Zhao^1^, Jing Zhou^1^, Huazhu Zhao^1^, Thamer Y. Mutter^2^, Xing Huang^1*^

1 Department of Microbiology, College of Life Sciences, Nanjing Agricultural University, Nanjing, Jiangsu, China

2 Department of Biology, College of Science, University of Anbar, Ramadi, Iraq

^*^Corresponding author: Xing Huang

1. mail: huangxing@njau.edu.cn





Figure 1: **(A)** the growth of maize in different concentrations of BSM-contaminated soil. **(B)** the growth of maize planted in BSM-polluted soil of 3 mg/kg in the different root-irrigation volumes of strain S113.
